# Aerobic exercise is associated with region-specific changes in volumetric, tensor-based, and fixel-based measures of white matter integrity in healthy older adults

**DOI:** 10.1016/j.ynirp.2022.100155

**Published:** 2023-01-04

**Authors:** Sarah E. Polk, Maike M. Kleemeyer, Nils C. Bodammer, Carola Misgeld, Johanna Porst, Bernd Wolfarth, Simone Kühn, Ulman Lindenberger, Sandra Düzel, Elisabeth Wenger

**Affiliations:** a Center for Lifespan Psychology, Max Planck Institute for Human Development, Berlin, Germany; b International Max Planck Research School on the Life Course (LIFE), Berlin, Germany; c Department of Sports Medicine, Charité – Universitätsmedizin Berlin and Humboldt Universität zu Berlin, Berlin, Germany; d Lise Meitner Group for Environmental Neuroscience, Max Planck Institute for Human Development, Berlin, Germany; e Max Planck UCL Centre for Computational Psychiatry and Ageing Research, Berlin, Germany

**Keywords:** Aerobic exercise, Cardiovascular fitness, Aging, White matter integrity, Diffusion-weighted imaging, Perceptual speed

## Abstract

White matter integrity and cognition have been found to decline with advancing adult age. Aerobic exercise may be effective in counteracting these declines. Generally, white matter integrity has been quantified using a volumetric measure (WMV) and with tensor-based parameters, such as fractional anisotropy (FA) and mean diffusivity (MD), the validity of which appears to be compromised in the presence of crossing fibers. Fixel-based analysis techniques claim to overcome this problem by yielding estimates of fiber density (FD), cross-section (FC), and their product (FDC) in multiple directions per voxel. In a sample of 61 healthy older adults (63–76 years old), we quantified changes in white matter integrity following an aerobic exercise intervention with the commonly used volumetric and tensor-based metrics (WMV, FA, MD) and with fixel-based metrics (FD, FC, FDC). We investigated the associations of changes in these white matter parameters with changes in cardiovascular fitness and Digit Symbol Substitution task (DSST) performance, a marker of perceptual speed. In line with previous findings, we observed maintained WMV in the corpus callosum of exercisers, and positive change-change correlations between WMV and fitness, and between WMV and perceptual speed. For FA and MD, group differences in change opposite to those hypothesized were found in the corpus callosum, posterior corona radiata, and superior longitudinal fasciculus at an uncorrected significance threshold. Likewise, regions in superficial WM in the prefrontal cortex showed group differences in FD and FDC change, uncorrected, with more positive change in controls and more negative change in exercisers. Finally, changes in FD and FDC were found to be inversely correlated to changes in fitness and DSST performance. The present results corroborate previous findings of WMV changes, but cast doubt on current physiological interpretations of both tensor-based and fixel-based indicators of white matter properties in the context of exercise intervention studies.

## Highlights

• At-home aerobic exercise improves cardiovascular fitness in older adults.
• Maintenance of corpus callosum volume is related to improved fitness and cognition.
• Indicators of frontal fiber density and cross-section decrease with exercise.
• Changes in fiber density indicator and cognition are inversely related.
• Results raise doubts about content validity of mean diffusivity and fractional anisotropy.

## 1 Introduction

The human brain undergoes a range of structural changes as senescence progresses. The deterioration of white matter integrity has been widely documented using various methods of quantification with both cross-sectional and longitudinal data (see reviews by Gunning-Dixon et al., 2009; Liu et al., 2016; Wassenaar et al., 2019). White matter volume (WMV), which is used to index macrostructural changes (i.e., general atrophy in aging), has been shown to increase until middle age and then quickly decrease starting around 60 years old (e.g., Bendlin et al., 2010; Geng et al., 2016; Raz et al., 2005). Diffusion tensor-derived measures of white matter integrity, which are used to approximate the direction and magnitude of the diffusion of water molecules through brain tissue, also show age-related changes; fractional anisotropy (FA), higher values of which are thought to indicate a higher degree of organization of white matter tracts, has been found to decrease with older age (e.g., Beck et al., 2021; Bendlin et al., 2010; Damoiseaux et al., 2009; Hsu et al., 2008; Kennedy and Raz, 2009; Lövdén et al., 2014), and this decrease has been shown to accelerate with age (Sexton et al., 2014). Mean diffusivity (MD), lower values of which are thought to indicate restricted movement of molecules and are thus typically interpreted as an indicator for denser tissue, has in turn been shown to increase with older age (e.g., Beck et al., 2021; Bendlin et al., 2010; Hsu et al., 2008), with this change again accelerating in older individuals (Sexton et al., 2014). The decreases in WMV and degradation of white matter microstructure indexed with FA and MD are thought to contribute to age-related cognitive decline, particularly in perceptual speed (see review by Bennett and Madden, 2014). For example, both FA and MD have been repeatedly shown to be related cross-sectionally to performance on tasks of perceptual speed (e.g., Bendlin et al., 2010; Hong et al., 2015; Kennedy and Raz, 2009; Kerchner et al., 2012; Turken et al., 2008).

Given this association between certain measures of white matter integrity and cognition at least cross-sectionally, efforts have been made to slow or even reverse the deterioration of white matter, with the aim of ameliorating age-related cognitive decline. In this regard, aerobic exercise (i.e., physical activity resulting in increased cardiovascular fitness) has been proposed as a lifestyle factor intervention. Overall, small but significant effects of physical fitness and physical activity on WMV have been found (see Sexton et al., 2016, for a meta-analysis). Mixed results have been found regarding microstructural integrity measured with FA and MD and its association with physical fitness and activity. Some cross-sectional studies have shown positive correlations with FA and negative correlations with MD (Gow et al., 2012; Johnson et al., 2012; Z. Liu et al., 2012; Tseng et al., 2013a), while others found no association (Burzynska et al., 2015; Marks et al., 2011; Tian et al., 2014). Longitudinally, one study showed that individual differences in fitness change were associated with change in FA and short-term memory (Voss et al., 2013), while another showed decreases in FA and no change in MD after exercise training (Clark et al., 2019). In a recent review, Erickson et al. (2019) concluded that there is moderate evidence supporting an association between aerobic exercise and cognition in a number of domains in older adults (also see Barha et al., 2017). Of note, an earlier review including twelve randomized controlled trials comparing aerobic exercise and a variety of control conditions found no evidence for an effect of aerobic exercise on cognition (Young et al., 2015). Additionally, there have been findings of the associations between white matter integrity and perceptual speed in both younger (Magistro et al., 2015) and older adults (Papp et al., 2014). However, the role of white matter integrity in the relationship between aerobic exercise and cognition is still unclear, as relatively few intervention studies have investigated this question (see Stillman et al., 2020) and even fewer have produced positive results (e.g., Voss et al., 2013).

Although many of the aforementioned studies used FA and MD as indicators of white matter integrity, the use of these diffusion tensor metrics has received criticism. The diffusion tensor model with which these metrics are derived is not fiber-specific, and is therefore not able to adequately distinguish between contributions from individual fibers to voxel-wise metrics in voxels with more complex multi-fiber geometry, such as crossing fibers (Raffelt et al., 2012, 2015, 2017), which poses a significant limitation as 60–90% of voxels within white matter have been estimated to contain crossing fibers (Jeurissen et al., 2013). In order to make up for this shortcoming, newer metrics have been developed using “fixels,” which represent specific fiber bundles within a voxel (Raffelt et al., 2017). Within each voxel, a fiber orientation distribution (FOD) of the fixels is computed using constrained spherical decomposition (Dhollander et al., 2016; Jeurissen et al., 2014). These FODs are then used to calculate apparent fiber density (FD), reflecting the density of fibers within a specific bundle, and fiber cross-section (FC), reflecting the diameter of a specific fiber bundle and commonly transformed to log(FC), as well as the product of FD and FC, fiber density and cross-section (FDC). These metrics are currently thought to be a more reliable measure of the physiological properties of white matter fibers than FA and MD. Supporting this, a combined histological and *ex vivo* MRI study in rodents (Rojas-Vite et al., 2019) found that histologically measured axonal density in both the optic nerve and optic chiasm correlated with FD in these regions estimated with constrained spherical deconvolution. Some studies have now also investigated the association of these fixel-based metrics with age. Choy et al. (2020) found that all three metrics showed widespread negative associations with age in a cross-sectional sample, particularly in anterior regions of the brain. Similarly, Kelley et al. (2021) found reduced FD, FC, and FDC in older adults versus younger adults, particularly in fronto-limbic areas. To our knowledge, the association between age-related decreases in the fixel-based metrics and cognition in healthy aging has not yet been explored, though a number of studies have found reduced values of FD, FC, and FDC in patients with Alzheimer's disease (Mito et al., 2018) and Parkinson's disease (Li et al., 2020; Rau et al., 2019; Zarkali et al., 2020). There has also been no investigation of the effects of an aerobic exercise intervention on the fixel-based metrics to date.

The current study thus aims to comprehensively investigate the relationship between aerobic exercise, various white matter integrity metrics, and perceptual speed. The current sample of older adults participated in a six-month intervention, either in an aerobic exercise group or an active control group. We expected to find (i) positive changes in WMV in exercisers as compared to controls. Given previous findings, we also expected to find (ii) increased FA values and decreased MD values as an effect of exercise, or overall decreases in FA and increases in MD, with no significant exercise effect. Thirdly, given the inverse association between age and fixel-based metrics, we expected to see (iii) an amelioration of age-related negative changes in FD, FC, and FDC as a result of aerobic exercise. We also explored correlations between cardiovascular fitness, white matter metrics, and performance on a task indexing perceptual speed. In this regard, we expected to see (iv) positive relationships between greater cardiovascular fitness, indicators of greater white matter integrity, and better performance on a perceptual speed task.

## 2 Materials and methods

### 2.1 Sample and study design

In the current set of exploratory analyses, we investigated the effects of aerobic exercise on white matter integrity in previously sedentary older adults by comparing individuals who participated in a physical training intervention group to those who did not engage in exercise. To isolate the effects of exercise, we used a subset of data from the AKTIV study, which investigated cognitive and physical exercise intervention effects in older adults. A full description of the study can be found in Wenger et al. (2022), and other findings using this sample on gray matter structural integrity and psychosocial functioning are reported respectively in Polk et al. (2022) and Düzel et al. (2022). We repeat all relevant details regarding subject recruitment, intervention design, and acquired measures for the current analyses here.

Healthy older adults from 63 to 78 years old were recruited if they met none of the following exclusion criteria: magnetic resonance imaging (MRI) contraindications; they could not meet the time requirements of the study; not right-handed; engaging in aerobic exercise more than once every two weeks; fluent in a language other than German or English, or fluent in more than two languages; or receiving medical treatment for Parkinson's, gout, rheumatism, heart attack, stroke, cancer, severe back problems, severe arrhythmia, severe chronic liver or kidney failure, severe disease of the hematopoietic system, mental illness (e.g., depression), or neurological disease (e.g., epilepsy, brain tumor).

Before the start of the intervention, participants first underwent a physical assessment including cardiopulmonary exercise testing (CPET) at the Charité – Universitätsmedizin Berlin, and were then invited to the Max Planck Institute for Human Development, Berlin for a baseline MRI session and cognitive testing (T1). Out of the 201 individuals invited to participate, 42 dropped out or were excluded due to existing medical conditions or claustrophobia in the scanner before the training. Participants trained at home in one of four intervention groups (active control, language, aerobic exercise, or combined language and aerobic exercise) for three months before being scanned a second time using the same sequences and completing the same cognitive battery at T2, and after a total of six months of at-home training, participants underwent MRI, cognitive testing, and a physical assessment a final time (T3). A further 17 participants dropped out during the training citing physical complaints (e.g., pain during exercise), disinterest, time constraints, or unspecified reasons, leaving a total of 142 participants who completed the study.

The ethics committee of the German Psychological Society (DGPs) approved the study and written informed consent was collected from all participants.

### 2.2 Interventions

In the current set of analyses, we focused on the isolated effects of aerobic exercise versus a sedentary lifestyle, without additional cognitive training, and compared the exercise-only group (EG) to the active control group (ACG).

Forty participants completed the study in the EG (mean age = 69.8 years, 50% females). Aerobic exercise was implemented with a stationary bicycle (DKN Ergometer AM-50) which was synchronized with a tablet (Lenovo TB2-X30L TAB) via Bluetooth. Using this tablet, participants could access their personalized interval training program, the initial level of which was determined at the first physical assessment (30 min at 25–140 Watts, M = 67.8, SD = 26.65). Participants were asked to exercise three to four times a week with no restrictions as to time of day. After each session, participants indicated their perceived exertion via the Borg Rating of Perceived Exertion Scale, which includes ratings from 6 (no exertion at all) to 20 (maximal exertion). If participants indicated a rating below 12 (too easy) or above 15 (too difficult), the intensity of the training could be adjusted remotely. Training intensity increased automatically approximately every two weeks by 3 min and three to four Watts. A collection of pre-selected literature was also available on the tablet, and participants were instructed to read at a slow pace for 15 min on days when they completed an exercise session, or for 45 min on days when they did not. In total, participants were expected to engage in study-related activity for approximately 45 min a day for at least six days each week. Finally, in-person group sessions of five to ten individuals each were conducted once a week, during which participants in the EG engaged in a stretching and toning course led by an external instructor. Adherence to the aerobic exercise intervention was defined as engaging in an average of 90 min of exercise a week for at least 21 weeks (≥1890 min total) with no pauses of longer than two weeks, as well as a slight steady increase in training difficulty over the course of the study, as was automatically implemented by the interval training application (see Table 1 in Results for adherence rates).

**Table 1:** Sample demographics and intervention specifics.

	Active control group	Exercise group
*n* completed intervention	35	40
*n* fully adhered	32 (91.4%)	29 (72.5%)
Age at baseline, M ± SD (range)	70.8 ± 3.93 (64.0–76.0)	70.2 ± 3.59 (63.9–76.9)
Sex, % of female participants	40.6	58.6
Years of education, M ± SD (range)	13.4 ± 3.15 (7–16)	13.0 ± 3.14 (7–16)
Total minutes spent in intervention, M ± SD (range)	4772 ± 1819.6 (2505–10858)	6554 ± 1222.9 (4098–9790)
Minutes spent reading, M ± SD (range)	4772 ± 1819.6 (2505–10858)	3381 ± 1143.3 (915–5855)
Minutes spent exercising, M ± SD (range)	–	3173 ± 410.4 (2556–3937)

*Note.* M = mean; SD = standard deviation. Age, sex, years of education, and total minutes spent in intervention were calculated among those participants who fully adhered to the intervention and were included in the current analyses.

Thirty-five participants completed the study in the ACG (mean age = 70.7 years, 40% females). They also received a tablet and were asked to read the selected literature for 45 min a day on at least six days of the week. In-person group sessions for participants in the ACG consisted of a book club, where groups discussed short stories led by external facilitators (http://shared-reading.de/). Adherence in the ACG was defined as at least 1890 total minutes of reading during the study (see Table 1 in Results for adherence rates).

### 2.3 Data acquisition

#### 2.3.1 Cardiovascular fitness

Cardiovascular fitness was measured as peak oxygen uptake, or VO_2_peak, relativized by body weight in kilograms, using CPET with a bicycle ergometer (Ergoselect 100k, Ergoline GmbH, Bitz, Germany) and the Quark Clinical-based Metabolic Cart using the standard Breath-by-Breath setup and the V2Mask (Hans Rudolph, Inc.).

#### 2.3.2 Magnetic resonance imaging

##### 2.3.2.1 Acquisition

MR images were acquired on a 3T Magnetom Tim Trio MRI scanner system (Siemens Medical Systems, Erlangen) using a 32-channel radiofrequency head coil. T_1_-weighted images were obtained using a 3D T_1_-weighted magnetization prepared gradient-echo (MPRAGE) sequence with the following parameters: repetition time (TR) = 2500 ms; echo time (TE) = 4.77 ms; inversion time (TI) = 1100 ms; flip angle = 7°; acquisition matrix = 256 × 256 × 192; 1 mm^3^ isotropic voxels; with the prescan normalize option and a 3D distortion correction for non-linear gradients; acquisition time = 9:20 min. Diffusion-weighted images were obtained with a single-shot diffusion-weighted spin-echo-refocused echo-planar imaging sequence with the following parameters: TR = 9700 ms; TE = 120 ms; 62 slices; FOV = 224 × 224 mm; a two-shell scheme was used for diffusion-weighting applying two *b*-values, 710 s/mm^2^ (30 directions) and 2850 s/mm^2^ (60 directions), with directions distributed over a whole sphere for each shell, plus ten non-diffusion-weighted images; GRAPPA acceleration factor = 2; 2 mm^3^ isotropic voxels; acquisition time = 16:41 min. Six additional images inverting the phase encoding direction were acquired without diffusion weighting; acquisition time = 1:29 min.

##### 2.3.2.2 Preprocessing and calculation of voxel-wise values

T_1_-weighted images were preprocessed using the longitudinal preprocessing pipeline with default parameters of the Computational Anatomy Toolbox 12 (CAT12, Structural Brain Mapping group, Jena University Hospital) in Statistical Parametric Mapping (SPM12, Institute of Neurology). Images were smoothed using an 8-mm full-width half-maximum (FWHM) standard Gaussian kernel.

Diffusion-weighted images were preprocessed using MRtrix (version 3.0_RC3; Tournier et al., 2019), FSL (FMRIB's Software Library, version 6.0.2; Jenkinson et al., 2012; Smith et al., 2004; Woolrich et al., 2009), and ANTS (version 2.2.0; Avants et al., 2010, 2011), following the Basic and Advanced Tractography with MRtrix for All Neurophiles (B.A.T.M.A.N.) tutorial (Tahedl, 2018). To create FA and MD maps, we followed the TBSS User Guide from FSL (https://fsl.fmrib.ox.ac.uk/fsl/fslwiki/TBSS/UserGuide; Smith et al., 2004, 2006). FA and MD maps were smoothed in SPM using a 4-mm FWHM standard Gaussian kernel for whole-brain analysis in SPM.

To calculate FD, log(FC), and FDC, we followed the “Fibre density and cross-section – Multi-tissue CSD” tutorial from the MRtrix3 documentation (https://mrtrix.readthedocs.io/en/latest/fixel_based_analysis/mt_fibre_density_cross-section.html; Tournier et al., 2019). In order to conduct repeated measures ANOVA on the fixel-based metrics in SPM, voxel-wise metrics were calculated for FD, log(FC), and FDC. For FD and FDC by summing the fixel-wise values across directions to calculate total FD and FDC per voxel; for log(FC), a weighted average was calculated across directions, where the log(FC) value in the direction with the greatest FD was weighted most heavily. These voxel-wise metrics were converted to Nifti format for longitudinal analysis in SPM. Finally, the FD, log(FC), and FDC maps were smoothed in SPM using a 10-mm FWHM standard Gaussian kernel.

More details regarding the preprocessing of MRI data and calculation of voxel-wise metrics can be found in the supplementary materials.

#### 2.3.3 Digit Symbol Substitution task

Perceptual speed was assessed with the Digit Symbol Substitution task (DSST; Wechsler, 1981). The DSST consists of a key to nine unique digit-symbol pairs, and rows of unpaired digits. Participants are asked to complete as many pairs as possible with the corresponding symbol within 90 s. Each correct answer is scored as 1, one incorrect answer is counted as 0, and after two consecutive incorrect answers, responses are no longer counted.

### 2.4 Statistical analyses

#### 2.4.1 Repeated measures ANOVA

To investigate group differences in change in VO_2_peak and DSST score, ANOVAs with time point as a within-subject factor (T1, T2, T3) and group as a between-subject factor (ACG, EG) were conducted, with age, sex, and years of education included as covariates. These were run using commands from the *rstatix* package (Kassambara, 2021) in R (R Core Team, 2021), version 4.1.2 (2021-11-01), using RStudio (RStudio Team, 2021), version 2021.09.2 + 382. *Post-hoc* t-tests were run using base R *stats* commands.

#### 2.4.2 Flexible factorial analysis investigating time-by-group interactions

To investigate group differences in change in the white matter metrics, flexible factorial models in SPM12 were used to compute voxel-wise statistics. This model, in contrast to the permutation-based models typically used in TBSS or FBA, can account for the fact that an individual's scans at different time points are not independent of one another. Smoothed WMV maps, smoothed FA and MD maps, and smoothed voxel-wise FD, log(FC), and FDC maps were entered into flexible factorial models with subject as a within-subject factor, time point as a within-subject factor (T1, T2, T3), and group as a between-subject factor (ACG, EG). Age, sex, and years of education were entered into the model as covariates of no interest. We tested for a time-by-group interaction to investigate whether changes across time points differed between groups. A threshold of *p* < .050 with correction for false discovery rate (FDR) at the peak-level was applied first, and if no significant clusters were revealed, a more liberal threshold of *p* < .001, uncorrected, was applied. In all cases, correction for non-isotropic smoothness was applied in CAT12 with a cluster extent threshold of *k* > 100. Missing data were excluded case-wise: no WMV data were missing, four cases were excluded from the diffusion tensor-derived and fixel-based metrics due to missing scans at one time point.

To investigate the directions of effects found, within-subject mean values at each time point were extracted from significant clusters using the REX: Response Exploration for Neuroimaging Datasets toolkit in MATLAB (Duff et al., 2007). Paired t-tests were conducted in R on these within-subject means to inspect within-group changes *post-hoc*.

#### 2.4.3 Correlations at baseline and change-change correlations

Finally, the relationships between VO_2_peak, white matter integrity metrics, and DSST score were investigated using Pearson correlations with the *Hmisc* R package (Harrell, 2021) and differences between correlations calculated within-group were examined with the *cocor* R package (Diedenhofen and Musch, 2015). Baseline correlations were calculated, as well as correlations between percent change from T1 to T3 in VO_2_peak, extracted white matter metrics from clusters showing group differences in change, and DSST score. Missing data points were excluded pair-wise. Correction for FDR was applied to baseline correlations and change-change correlations separately.

## 3 Results

A description of the sample can be found in Table 1. Participants who did not meet compliance criteria were excluded and one further participant in the EG was excluded due to technical difficulties. This resulted in *n*_ACG_ = 32 and *n*_EG_ = 29 included in the analyses. A *post-hoc* sensitivity analysis using G*Power (version 3.1.9.6) indicated that, with *α* = 0.05, 1 – *β* = 0.95, and a study design with two groups and three time points, a sample size of *N* = 61 could reliably capture time-by-group interaction effects with a critical *F* ≥ 3.073 and correlations with a coefficient of *r* ≥ 0.438.

### 3.1 Group differences in cardiovascular fitness change

Means and standard deviations of VO_2_peak at T1 and T3 for each group are reported in Table 2. A repeated measures ANOVA including age, sex, and years of education as covariates revealed a significant time-by-group interaction in VO_2_peak, *F*(1, 53) = 6.091, *p* = .017, Hedge's *g* = 0.009. *Post-hoc* pairwise t-tests indicated a significant increase in VO_2_peak within exercisers, *t*(28) = 4.959, *p* < .001, with a mean percent change of 12.8% (SE = 2.28), but not within controls, *t*(29) = 1.279, *p* = .211, with a mean percent change of 3.7% (SE = 2.22).

**Table 2:** Means and standard deviations of variables of VO_2_peak, Digit Symbol Substitution task score, and white matter metrics extracted from clusters showing significant time-by-group interactions*.*

	Active control group			Exercise group		
Measure	T1	T2	T3	T1	T2	T3
VO_2_peak (mL/kg/min)	23.5 ± 6.01	–	24.4 ± 6.52	22.9 ± 6.03	–	25.5 ± 6.21
DSST score	46.8 ± 8.41	47.3 ± 7.76	47.5 ± 8.83	46.1 ± 10.76	44.7 ± 10.65	49.1 ± 11.27

Metric	Region	Active control group			Exercise group		
		T1	T2	T3	T1	T2	T3
WMV	rACR/genu	0.568 ± 0.0948	0.564 ± 0.0945	0.554 ± 0.0968	0.536 ± 0.0892	0.539 ± 0.0893	0.534 ± 0.0896
	splenium	0.533 ± 0.0836	0.529 ± 0.0843	0.521 ± 0.0837	0.511 ± 0.0759	0.512 ± 0.0750	0.511 ± 0.0751
FA	genu	0.334 ± 0.0315	0.343 ± 0.0388	0.346 ± 0.0365	0.352 ± 0.0366	0.353 ± 0.0394	0.344 ± 0.0364
MD	rPCR/splenium	9.12 × 10^−04^ ± 9.06 × 10^−05^	9.03 × 10^−04^ ± 8.19 × 10^−05^	9.01 × 10^−04^ ± 8.55 × 10^−05^	8.92 × 10^−04^ ± 5.24 × 10^−05^	8.98 × 10^−04^ ± 5.42 × 10^−05^	9.04 × 10^−04^ ± 5.44 × 10^−05^
	rSLF	8.32 × 10^−04^ ± 5.73 × 10^−05^	8.21 × 10^−04^ ± 5.58 × 10^−05^	8.17 × 10^−04^ ± 6.31 × 10^−05^	8.13 × 10^−04^ ± 4.93 × 10^−05^	8.21 × 10^−04^ ± 5.09 × 10^−05^	8.23 × 10^−04^ ± 5.00 × 10^−05^
FD	dmPFC	0.036 ± 0.0036	0.037 ± 0.0033	0.038 ± 0.0039	0.037 ± 0.0038	0.036 ± 0.0037	0.035 ± 0.0032
	dlPFC	0.010 ± 0.0028	0.011 ± 0.0030	0.012 ± 0.0036	0.010 ± 0.0022	0.010 ± 0.0021	0.010 ± 0.0025
FDC	dmPFC	0.048 ± 0.0088	0.048 ± 0.0073	0.050 ± 0.0084	0.048 ± 0.0084	0.047 ± 0.0086	0.046 ± 0.0069
	dlPFC	0.012 ± 0.0039	0.012 ± 0.0043	0.014 ± 0.0049	0.011 ± 0.0027	0.011 ± 0.0026	0.011 ± 0.0028

*Note*. DSST = Digit Symbol Substitution task; WMV = white matter volume; FA = fractional anisotropy; MD = mean diffusivity (mm^2^/s); FD = fiber density; FDC = fiber density and cross-section. rACR = right anterior corona radiata; rPCR = right posterior corona radiata; rSLF = right superior longitudinal fasciculus; dmPFC = dorsomedial prefrontal cortex; dlPFC = dorsolateral PFC.

### 3.2 Group differences in white matter integrity changes

#### 3.2.1 Whole-brain white matter volume

**Fig. 1:**
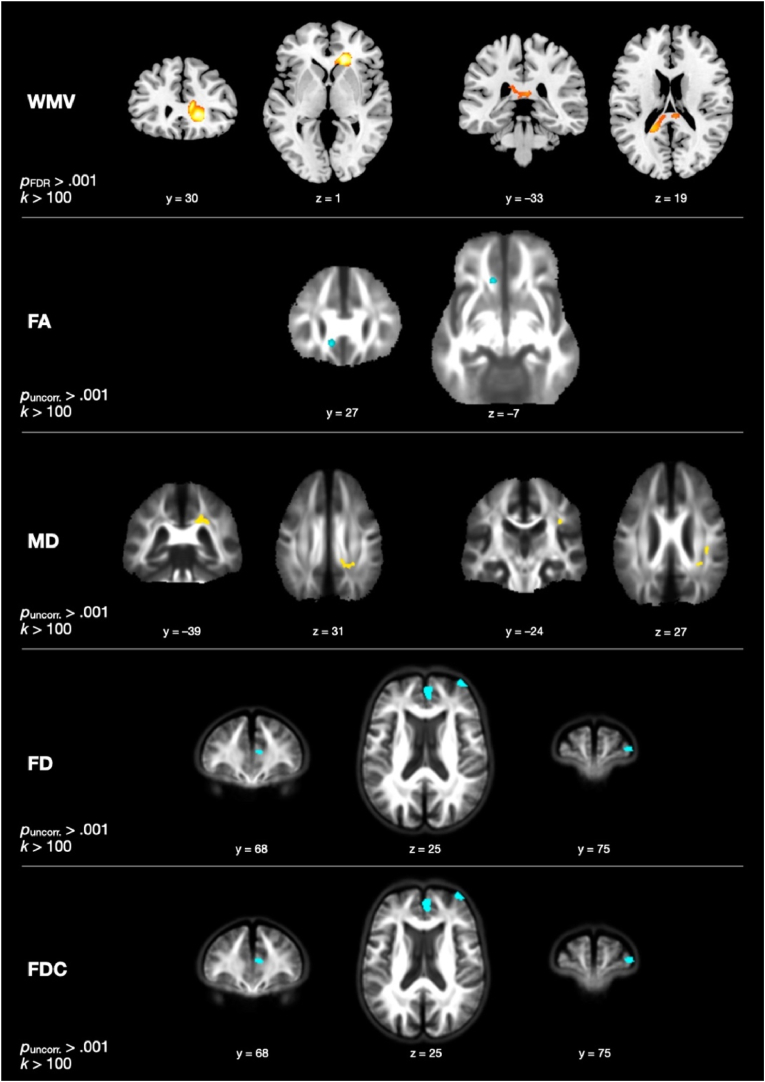
Group differences in change in white matter volume (WMV), fractional anisotropy (FA), mean diffusivity (MD), fiber density (FD), fiber density and cross-section (FDC). Yellow/orange-colored clusters represent more positive changes in exercisers than controls, cyan-colored clusters represent more negative changes in exercisers than controls. WMV, FA, and MD are calculated and displayed in MNI space, whereas FD and FDC are calculated and displayed in a study-specific space. (For interpretation of the references to color in this figure legend, the reader is referred to the Web version of this article.)

The contrast investigating group differences in change in WMV, controlling for age, sex, and years of education, revealed two significant clusters at *p*_FDR_ < .050 with a cluster size threshold of *k* > 100 (see Fig. 1). One cluster was found in the right anterior corona radiata extending into the genu of the corpus callosum (rACR/genu; 1000 voxels; peak *F* = 33.90; peak voxel: *x* = 16, *y* = 29, *z* = 2); the EG showed no significant change in mean WMV within this cluster from T1 to T3, *t*(28) = −1.655, *p* = .109, while controls decreased significantly, *t*(31) = −7.682, *p* < .001. The other cluster was localized in the splenium of the corpus callosum (542 voxels; peak *F* = 20.86; peak voxel: *x* = −21, *y* = −45, *z* = 16); in this cluster, the EG again showed no significant change, *t*(28) = −0.170, *p* = .866, while the ACG showed a significant decrease, *t*(31) = −6.581, *p* < .001.

#### 3.2.2 Whole-brain fractional anisotropy

No clusters showing significant time-by-group effects in FA were found at a threshold of *p*_FDR_ < .050, *k* > 100. At *p*_uncorrected_ < .001, one cluster was revealed (see Fig. 1). In this cluster in the left part of the genu of the corpus callosum (136 voxels; peak *F* = 23.35; peak voxel: *x* = −10, *y* = 27, *z* = 8), the EG showed a significant decrease, *t*(28) = −4.229, *p* < .001, while the ACG showed a significant increase in FA, *t*(27) = 5.149, *p* < .001.

#### 3.2.3 Whole-brain mean diffusivity

Regarding changes in MD, no clusters survived the threshold of *p*_FDR_ < .050, but two clusters showing a significant time-by-group interaction were revealed at the more lenient threshold of *p*_uncorrected_ < .001 (see Fig. 1). One cluster was found in the right posterior corona radiata extending into the splenium of the corpus callosum (rPCR/splenium; 358 voxels; peak *F* = 18.88; peak voxel: *x* = 26, *y* = −38, *z* = 28), in which the EG showed a significant increase in mean MD, *t*(28) = 4.530, *p* < .001, and the ACG showed a significant decrease, *t*(27) = – 2.663, *p* = .013. The other cluster was found in the right superior longitudinal fasciculus (rSLF; 100 voxels; peak *F* = 18.38; peak voxel: *x* = 34, *y* = −25, *z* = 28), in which the EG again showed a significant increase in mean MD, *t*(28) = 2.450, *p* = .021, and the ACG showed a significant decrease, *t*(27) = −3.458, *p* = .002.

#### 3.2.4 Whole-brain fiber density

Testing for time-by-group interactions in FD revealed two significant clusters larger than 100 voxels at *p*_uncorrected_ < .001, neither of which survived FDR correction (see Fig. 1). One cluster was localized in the right dorsomedial prefrontal cortex (dmPFC, 338 voxels; peak *F* = 23.73; peak voxel: *x* = 3, *y* = 65, *z* = 26 in study-specific space), and another was seen in the right dorsolateral prefrontal cortex (dlPFC, 105 voxels; peak *F* = 16.83; peak voxel: *x* = 39, *y* = 78, *z* = 25 in study-specific space). In the dmPFC, the EG showed a significant decrease in mean FD from T1 to T3, *t*(28) = −3.472, *p* = .002, while the ACG showed a significant increase, *t*(27) = 4.255, *p* < .001. In the dlPFC, the EG showed no change in mean FD, *t*(28) = −1.556, *p* = .131, and the ACG showed a significant increase, *t*(27) = 3.545, *p* = .001.

#### 3.2.5 Whole-brain fiber cross-section

No significant clusters were revealed when testing for group differences in change in log(FC), either at the initial threshold of *p*_*FDR*_ < .050 or the more liberal threshold of *p*_uncorrected_ < .001.

#### 3.2.6 Whole-brain fiber density and cross-section

Finally, two clusters were found in which there were significant group differences in change in FDC at *p*_uncorrected_ < .001 (see Fig. 1), both of which almost entirely overlapped with those found in FD: one in the right dmPFC (386 voxels; peak *F* = 24.79; peak voxel: *x* = 3, *y* = 65, *z* = 26 in study-specific space) and one in the right dlPFC (130 voxels; peak *F* = 16.97; peak voxel: *x* = 38, *y* = 78, *z* = 25 in study-specific space). These clusters did not survive FDR correction either. The pattern of within-group change mirrored that seen in FD: in the dmPFC, the EG decreased significantly from T1 to T3, *t*(28) = −3.208, *p* = .003, while the ACG increased, *t*(27) = 3.931, *p* < .001. In the dlPFC, the EG showed no significant change, *t*(28) = −1.705, *p* = .099, and the ACG showed a significant increase, *t*(27) = 3.399, *p* = .002.

Means and standard deviations of extracted mean values from each cluster showing a significant time-by-group interaction can be found in Table 2.

### 3.3 Cognition

DSST score means and standard deviations are reported in Table 2. No significant time-by-group effects were found in DSST when controlling for age, sex, and years of education, *F*(2, 102) = 2.696, *p* = .072, Hedge's *g* = 0.010. *Post-hoc* pairwise t-tests indicated a significant increase in DSST score within the EG, *t*(26) = 2.213, *p* = .036, but not within the ACG, *t*(29) = −0.163, *p* = .872.

### 3.4 Correlations

Baseline correlations between VO_2_peak, DSST, and extracted mean values within each of the clusters showing significant time-by-group differences, as well as correlations between percent change in white matter metrics, all corrected for FDR, can be found in Table 3.

**Table 3:** Correlation coefficients of baseline and percent change correlations between VO_2_peak, Digit Symbol Substitution task score, and white matter metrics extracted from clusters showing significant time-by-group interactions.

Baseline	1	2	3	4	5	6	7	8	9	10
1. VO_2_peak	–									
2. DSST	.11	–								
3. WMV rACR/genu	.40*	.17	–							
4. WMV splenium	.33*	.01	.77*	–						
5. FA genu	−.01	.14	.20	.20	–					
6. MD rPCR/splenium	−.05	−.03	−.16	−.18	−.53*	–				
7. MD rSLF	−.32*	−.11	−.34*	−.21	−.38*	.40*	–			
8. FD dmPFC	−.07	.05	.28	.03	.43*	−.22	−.32*	–		
9. FD dlPFC	.25	.09	.13	.10	.14	−.08	−.26	.30	–	
10. FDC dmPFC	.07	.01	.70*	.48*	.31*	−.22	−.31*	.78*	.25	–
11. FDC dlPFC	.31*	.04	.46*	.38*	.18	−.09	−.27	.40*	.88*	.51*

Percent change	1	2	3	4	5	6	7	8	9	10

1. VO_2_peak	–									
2. DSST	.02	–								
3. WMV rACR/genu	.18	.34*	–							
4. WMV splenium	.33*	.26	.65*	–						
5. FA genu	−.23	.01	−.21	−.26	–					
6. MD rPCR/splenium	.17	.20	.18	.32*	−.39*	–				
7. MD rSLF	.06	−.14	.13	.10	−.42*	.47*	–			
8. FD dmPFC	−.32*	−.12	−.38*	−.41*	.46*	−.41*	−.28	–		
9. FD dlPFC	−.09	−.31	−.17	−.24	.37*	−.52*	−.46*	.31*	–	
10. FDC dmPFC	−.30*	−.12	−.36*	−.40*	.41*	−.38*	−.26	.98*	.26	–
11. FDC dlPFC	−.09	−.30	−.19	−.26	.36*	−.50*	−.46*	.30*	1.00*	.25

*Note*. DSST = Digit Symbol Substitution task; WMV = white matter volume; FA = fractional anisotropy; MD = mean diffusivity; FD = fiber density; FDC = fiber density and cross-section; rACR = right anterior corona radiata; rPCR = right posterior corona radiata; rSLF = right superior longitudinal fasciculus; dmPFC = dorsomedial prefrontal cortex; dlPFC = dorsolateral PFC.

* Significant at *p*_FDR_ < .05.

Regarding correlations with percent change in cardiovascular fitness, a positive correlation was found between percent change in VO_2_peak and percent change in WMV in the splenium of the corpus callosum, *r*(57) = 0.33, *p*_FDR_ = .029. Negative correlations were found between percent change in VO_2_peak and percent change in FD in the dmPFC, *r*(55) = −0.32, *p*_FDR_ = .034, and percent change in FDC in the dmPFC, *r*(55) = −0.30, *p*_FDR_ = .047. No differences between within-group correlation coefficients were found.

**Fig. 2:**
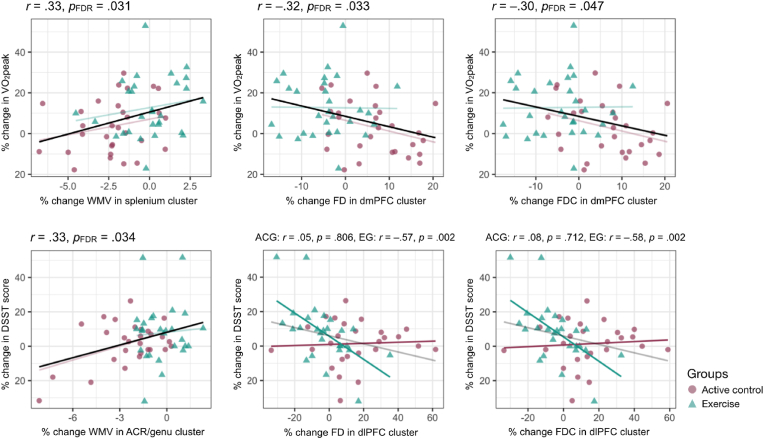
Significant correlations between percent change in VO_2_peak, white matter integrity metrics extracted from clusters with significant time-by-group interactions, and Digit Symbol Substitution task score. Overall correlations are shown in opaque black for those correlations that do not show significant group differences (top row: left, center, right; bottom row: left); for these, group-wise correlations are shown as transparent color-coded lines. Correlations that differ significantly between groups are represented by opaque color-coded lines (bottom row: center, right); for these, overall correlations are shown as transparent black lines. WMV = white matter volume; FD = fiber density; FDC = fiber density and cross-section; dmPFC = dorsomedial prefrontal cortex; DSST = Digit Symbol Substitution task; ACR = anterior corona radiata; dlPFC = dorsolateral prefrontal cortex. (For interpretation of the references to color in this figure legend, the reader is referred to the Web version of this article.)

Correlations with percent change in DSST score were also detected. A positive correlation was found with percent change in WMV in the rACR/genu of the corpus callosum, *r*(51) = 0.35, *p*_FDR_ = .026. Weak negative correlations with percent change in FD and FDC in the dlPFC were also found, *r*(49) = −0.31, *p*_uncorrected_ = .014 and *r*(49) = −0.31, *p*_uncorrected_ = .023, though these did not survive FDR correction, *p*_FDR_ = .051 and *p*_FDR_ = .053, respectively. No group difference was found in the change-change correlation between DSST and WMV. The correlation between percent change in DSST score and percent change in FD was significantly different between groups, Fisher's *z* = 2.399, *p* = .016; the EG showed a significant negative correlation, *r*(25) = −0.57, *p* = .002, while no significant association was found within the ACG, *r*(24) = 0.05, *p* = .806. The correlation between percent change in DSST score and percent change in FDC was also significantly different between groups, Fisher's *z* = 2.537, *p* = .011; the EG showed a significant negative correlation, *r*(25) = −0.58, *p* = .002, while no significant association was found within the ACG, *r*(24) = 0.08, *p* = .712. See Fig. 2 for visualization of significant correlations between percent changes in variables of interest.

Of note, removing the visual outlier in percent change in VO_2_peak did not affect the significance of the results: percent change in WMV in the cluster found in the splenium and percent change in VO_2_peak were positively correlated, *r* = 0.33, *p*_uncorrected_ = .010; percent change in FD in the cluster found in the dmPFC and percent change in VO_2_peak were negatively correlated, *r* = −0.35, *p*_uncorrected_ = .009; and percent change in FD in the cluster found in the dmPFC and percent change in VO_2_peak were negatively correlated *r* = −0.32, *p*_uncorrected_ = .017. Similarly, removing the two visual outliers in percent change in DSST score resulted in a correlation of *r* = 0.33, *p*_uncorrected_ = .015, between percent change in WMV in the cluster found in the rACR/genu and percent change in DSST score, a within-controls correlation of *r* = 0.05, *p*_uncorrected_ = .806, and a within-exercisers correlation of *r* = −0.48, *p*_uncorrected_ = .015, between percent change in FD in the dlPFC cluster and percent change in DSST score, and a within-controls correlation of *r* = 0.08, *p*_uncorrected_ = .712, and a within-exercisers correlation of *r* = −0.50, *p*_uncorrected_ = .011, between percent change in FDC in the dlPFC cluster and percent change in DSST score.

No significant correlation was found between change in VO_2_peak and change in DSST score, *r*(51) = 0.02, *p*_FDR_ = .882.

Finally, given that we found an effect of aerobic exercise on white matter integrity and in order to better understand the relationships between the white matter metrics themselves, *post-hoc* Pearson correlations were calculated using the *Hmisc* R package (Harrell, 2021). We extracted mean FA, MD, FD, log(FC), and FDC from the most robust clusters showing group-by-time effects, namely those clusters in the rACR/genu and splenium in which the EG showed no change while the ACG showed decreases in WMV, using REX. These results can be found in Table 4.

**Table 4:** Correlation coefficients of baseline and percent change correlations between white matter metrics extracted from clusters showing robust time-by-group interactions.

Baseline	rACR/genu cluster					Splenium cluster				
	1	2	3	4	5	1	2	3	4	5
1. WMV	–					–				
2. FA	.38*	–				.41*	–			
3. MD	−.23	−.73*	–			−.40*	−.57*	–		
4. FD	.26	.61*	−.51*	–		.41*	.62*	−.45*	–	
5. log(FC)	.91*	.24	−.15	.28*	–	.76*	.17	−.52*	.23	–
6. FDC	.76*	.50*	−.39*	.75*	.84*	.73*	.53*	−.64*	.79*	.77*

Percent change	rACR/genu cluster					Splenium cluster				

1	2	3	4	5	1	2	3	4	5
1. WMV	–					–				
2. FA	.05	–				−.13	–			
3. MD	−.28	−.35	–			.25	−.55*	–		
4. FD	−.01	−.06	−.08	–		.04	−.07	.14	–	
5. log(FC)	−.22	.07	.08	.25	–	.06	.09	−.03	−.01	–
6. FDC	−.01	−.06	−.08	1.00*	.25	.04	−.08	.15	1.00*	−.01

*Note.* rACR = right anterior corona radiata; WMV = white matter volume; FA = fractional anisotropy; MD = mean diffusivity; FD = fiber density; FDC = fiber density and cross-section; log(FC) = logarithm of fiber cross-section.* significant at *p*_FDR_ < .05.

## 4 Discussion

This study investigated the effects of aerobic exercise on several white matter integrity metrics including (i) WMV, derived from voxel-based morphometry, (ii) FA and MD, derived using diffusion tensor models, and (iii) FD, log(FC), and FDC, derived using fixel-based analyses. In particular, given the known weaknesses of diffusion tensor modeling, we were interested in whether fixel-based analysis would be better suited to capturing exercise-induced changes in white matter integrity in a sample of healthy older adults. We also looked at the associations between the changes in each of the metrics used to capture white matter integrity. Finally, we looked at (iv) correlations with change in cardiovascular fitness and change in a cognitive task indexing perceptual speed.

Participants in the aerobic exercise group engaged in at-home interval training on a stationary bike for three to four days a week for six months, leading to an increase in cardiovascular fitness (VO_2_peak) compared to active control participants. This indicates that at-home aerobic exercise that is personalized to the individual is an effective intervention for cardiovascular fitness in older adults. This finding is discussed in greater detail in Polk et al. (2022).

We found evidence of exercise-induced maintenance of WMV in the current sample, substantiating our first hypothesis. Namely, we found two clusters, one in the rACR extending into the genu of the corpus callosum, and one in the splenium of the corpus callosum, in which change over six months was significantly different between the group engaging in regular aerobic exercise and the sedentary group. This finding is consistent with several cross-sectional studies which found effects of physical activity on WMV (Benedict et al., 2013; Erickson et al., 2007; Gow et al., 2012; Ho et al., 2011; Tseng et al., 2013b). Furthermore, we were able to replicate findings from a previous six-month intervention study in which older adults in a similar age range (60–79 years) participated in either a supervised aerobic exercise group or a nonaerobic stretching and toning group (Colcombe et al., 2006). This study also found more positive change in WMV in anterior white matter, namely in the genu of the corpus callosum as an effect of exercise. Our study differs from the previous in that the current design implemented a flexible, at-home training regimen, rather than in-lab exercise sessions under supervision from a personal trainer. This indicates that at-home exercise, which may be more accessible for older adults, has a similar impact on WMV as supervised, in-lab exercise.

Additionally, change in WMV in the splenium was correlated with change in cardiovascular fitness, with more positive change in VO_2_peak associated with reduced loss of WMV, supporting our fourth hypothesis. This effect seemed to be general, as no difference between within-group correlation coefficients was detected, indicating that this relationship was specifically not exercise-induced. However, the significant increase in VO_2_peak seen in the EG over the course of the intervention may have contributed to the maintenance of WMV in the splenium. This is consistent with a number of cross-sectional (e.g., Erickson et al., 2007; Ho et al., 2011) and longitudinal (e.g., Colcombe et al., 2006) studies finding associations of physical activity, aerobic exercise, and cardiovascular fitness with WMV in both frontal and parietal areas. Finally, change in WMV in the rACR/genu of the corpus callosum was correlated with change in DSST score, with reduced loss of volume being associated with a greater increase in score from T1 to T3, further supporting our fourth hypothesis. This corroborates cross-sectional findings of anterior corpus callosum size being associated with performance on the DSST (Fling et al., 2011). Again, this effect seemed to be general, with no group difference in correlation, but given the preservation of WMV in the EG compared to the ACG, as well as the significant increase in DSST score among exercisers, a causal relationship between aerobic exercise, anterior corpus callosum WMV, and performance on the DSST seems plausible.

The findings of the diffusion tensor model-derived metrics, FA and MD, did not support our hypothesis that exercise should mitigate decreasing FA and increasing MD. In the current sample, we found that FA decreased within exercisers and increased within controls in the left part of the genu of the corpus callosum. In the rPCR/splenium as well as in the rSLF, MD values in the EG increased while they decreased in the ACG. Notably, the clusters showing group-by-time interactions in FA and MD did not survive correction for multiple testing, so we only interpret these results cautiously in the following. In part, this finding corroborates earlier work by Clark et al. (2019), who found widespread decreases in FA and increases in MD in a group of 57- to 86-year-old individuals who participated in supervised aerobic exercise in the form of walking for six months, although this report did not include comparisons with a control group. Additionally, Voss et al. (2013) found no group-level effects of one year of supervised aerobic walking, compared to a flexibility, toning, and balance condition, on white matter integrity as measured by whole-brain FA in older adults aged 55–80 years. Our study differs from both the Clark et al. (2019) and Voss et al. (2013) studies in the type and context of aerobic exercise implemented; while both of the previously mentioned studies administered a supervised, in-lab aerobic walking paradigm, the current design used an at-home stationary bicycle-based interval training. Future studies may consider how different types of aerobic exercise (e.g., walking vs. biking, at-home vs. supervised in-lab) could have a different impact on the white matter metrics of FA and MD in older adults in order to more systematically understand their patterns of change in the context of aging and aerobic exercise.

Regarding this finding within the scope of aging, in a study comparing whole-brain FA values between younger and older adults, a number of regions, including the cingulum bundle, in which the current analyses showed a decrease in FA among exercisers but not controls, were found to have greater FA values in older adults as compared to younger adults (Kelley et al., 2021). The increases in FA found in this area within controls may therefore be consistent with age-related decline, and the decrease in FA induced by aerobic exercise in older adults would then be indicative of a protective effect of exercise. Regarding the cluster of MD in the rPCR/splenium of the corpus callosum, in which the EG increased while the ACG increased, it may be important to consider both the biological underpinnings of MD here, as well as the specific location of this cluster. Within regions where white matter tracts are highly unidirectional, such as within the body of the corpus callosum, FA and MD values may accurately map onto a number of factors indicating white matter integrity, including fiber coherence, fiber diameter and density, and myelination (Basser and Pierpaoli, 1996; Beaulieu, 2002; Pierpaoli and Basser, 1996). However, the diffusion tensor model has known shortcomings in the face of crossing fibers (Raffelt et al., 2012, 2015, 2017). Indeed, Kelley et al. (2021) found strong negative voxel-wise correlations between FA and a measure of multi-fiber complexity, an index of the number of crossing fibers, throughout the brain. In the current analyses, one of the clusters in which MD showed a time-by-group effect was located at the intersection of two major white matter tracts: the corpus callosum and the corona radiata. The increase of MD in this area may thus be representative of an increase in the prominence of crossing fibers in this area, which is plausibly beneficial at the convergence of major tracts. This could also explain an increase in WMV (although not in an overlapping cluster, but located in a similar area in the opposite hemisphere), as an increase in myelination in multiple directions would increase the proportion of white matter found within a voxel. In support of this interpretation, we found a positive correlation between percent change in WMV in the splenium and percent change in MD in the rPCR/splenium of the corpus callosum (see Table 3). Ultimately, one should consider the drawbacks of the diffusion tensor model when interpreting age- and exercise-induced patterns of change in FA and MD.

Finally, FD and FDC findings were quite similar to one another, which is unsurprising as FDC is simply a linear combination (i.e., the product) of FD and FC. Two clusters in the PFC were found, one in the dmPFC and one in the dlPFC, however, the direction of the effects again went in the opposite direction of that which we hypothesized given previous findings regarding age-related effects on the fixel-based parameters (Choy et al., 2020; Kelley et al., 2021). Namely, significant decreases in both FD and FDC were observed in the dmPFC cluster within exercisers, while controls showed increases. In the dlPFC cluster, the EG showed no change, however the ACG still showed significantly more positive change than the EG. This seems to indicate that the density as well as combined density and cross-section of fiber bundles decreased as an effect of aerobic exercise. Again, these clusters did not survive correction for multiple testing and are interpreted with caution here.

Interestingly, in the dmPFC, change in both FD and FDC values were negatively correlated with change in VO_2_peak, with decreases in fitness being associated with greater increases in FD and FDC. This effect seemed to be unrelated to the exercise intervention, as the group-wise correlations were not significantly different. Altogether, this seems to suggest that increases in FD and FDC in this specific region are associated with age-related decline, and that aerobic exercise and improved cardiovascular fitness may ameliorate this decline. Notably, the correlation with change in VO_2_peak was also not significant within either group, which could indicate a lack of power, and the overall correlation should be interpreted with caution, given the group differences in both FD and FDC changes, as well as in VO_2_peak change. In the dlPFC cluster, changes in FD and FDC were found to be weakly negatively correlated with change in DSST score, and these correlations were significantly different between groups, with the EG showing a significant negative correlation and the ACG showing no association. This suggests that, on a functional level, lower levels of FD and FDC in this area in the dlPFC could be beneficial in aging, with greater exercise-induced declines being associated with greater improvement in performance on a task requiring a range of cognitive processes, including perceptual speed and executive function.

Together, these findings suggest that the directionality of age-related changes as well as exercise-induced changes may be region-specific in terms of functional adaptivity. Notably, both clusters found in the current analyses of FD and FDC were localized at the border between white matter and gray matter, in what is known as superficial white matter. Studies using FA and MD have found an inverse relationship between superficial FA and age (Nazeri et al., 2015), as well as cross-sectional associations between diffusion tensor metrics and cognitive function (Reginold et al., 2016). However, there has been little to no investigation of region-specific associations between age, cognition, and structural integrity in these superficial white matter areas due to methodological limitations (Kirilina et al., 2020).

Finally, we were interested in the relationships between the various white matter metrics themselves, especially across the different analysis techniques. We therefore extracted all the metrics of interest from the most robust clusters showing group-by-time interaction effects, namely the cluster in the rACR extending into the genu of the corpus callosum and the cluster in the splenium of the corpus callosum. At baseline, as expected, WMV was positively correlated with FA and the fixel-based metrics, and negatively with MD; FA was positively correlated with the fixel-based metrics, MD was negatively correlated with FD and FDC, and FA and MD were negatively correlated; and FD, FDC, and log(FC) were positively correlated with one another. This indicates a linear relationship among the metrics when observing them cross-sectionally. However, we observed no significant correlations among percent changes in these metrics, with the exceptions of FA and MD, which were negatively correlated, and FD and FDC, which were almost perfectly positively correlated. The absence of significant change-change correlations could indicate that the variance in change measured with these metrics was not different from zero, restricting our ability to measure change-change correlations; future methodological studies should aim to test how reliably changes in these metrics, particularly in the newer fixel-based metrics, can be captured. Alternatively, if the absence of significance is indicative of a true null result, this could mean that while within a certain analysis pipeline, changes across individual metrics are associated with one another, each of the analysis pipelines captures a different aspect of white matter integrity, and each of these aspects of white matter integrity changes in a differentiated manner.

While WMV is a more general measure of white matter integrity that specifically does not account for underlying anatomical structures (e.g., cellular structures, fibers or fiber bundles), the diffusion tensor-based and fixel-based metrics aim to capture microstructural white matter integrity and changes therein. Given the drawbacks of the diffusion tensor model discussed previously that potentially limit their validity to measure microstructural integrity in a majority of white matter, it may be beneficial to devote future research to more fully understanding the anatomical underpinnings of age-related decreases in FD, FC, and FDC in humans. As the fixel-based metrics were designed to capture changes in fiber bundles, the use of these metrics of course does not preclude the use of e.g., WMV to understand more general atrophy due to aging, and indeed these two methods could be used complementarily to more deeply investigate how aging affects the brain, and in turn, how age-related deterioration of white matter can be reversed through interventions such as aerobic exercise.

Altogether, the findings of the current study provide evidence of an effect of aerobic exercise on WMV, tensor-based FA and MD, and fixel-based FD and FDC in older adults, and show that increases in WMV in the corpus callosum and reductions FD and FDC in superficial WM in the PFC are related to increased fitness as well as improvement on a cognitive task indexing perceptual speed.

### 4.1 Limitations

The current study has a number of limitations that warrant mentioning. First, given the interventional nature of the study, the sample was only moderately sized. This may have reduced our power to find robust effects that would survive correction for multiple comparisons. Future studies should aim to replicate the current findings with larger sample sizes. Second, the sample in the current study is relatively homogeneous: participants were recruited from an area with relatively high socioeconomic status, they were well-educated on average, and indicated no major health problems, despite not engaging in physical activity on a regular basis. Thus, there could be other protective factors at play apart from aerobic exercise. Future studies should aim to generalize the findings of the current study in more heterogeneous samples. Finally, regarding the white matter findings using diffusion tensor model- and fixel-based metrics, the clusters in which groups differed in change that were found at threshold of *p*_uncorrected_ < .001 did not survive correction for false discovery rate, so these results should be interpreted with caution, as previously mentioned. Furthermore, both clusters of FD and FDC change in the dmPFC and dlPFC were localized at the edge of the cortex in superficial white matter. These fiber bundles are more complicated to study than deep white matter, given their proximity to gray matter, and may also be more susceptible to noise during MR acquisition, leading to difficulties in the estimation of certain WM tracts (Guevara et al., 2020; Kirilina et al., 2020; Reveley et al., 2015). Additionally, the current study used *b*-values of 710 s/mm^2^ and 2850 s/mm^2^ for multi-shell analyses. However, a recent study investigating the impact of different *b*-values on the estimation of FD in a sample of children and adolescents (8–18 years old) found that multi-shell schemes including higher *b*-values of 4000 s/mm^2^ or even 6000 s/mm^2^ improved the sensitivity of tract-specific FD to age associations (Genc et al., 2020). Future research interested in associations between age and WM integrity measured with fixel-based metrics may therefore consider acquiring MR data with sequences that specifically target superficial WM, or with diffusion-weighted imaging using higher *b*-values.

## 5 Conclusion

The current study investigated the effects of aerobic exercise on white matter structure in older adults using voxel-based morphometry, the diffusion tensor model, and fixel-based metrics. We found strong evidence that aerobic exercise is protective of WMV in the corpus callosum, replicating previous findings. We also found that these changes positively correlated with both changes in cardiovascular fitness and changes in performance on a cognitive task indexing perceptual speed. We also found weak evidence of a decrease in FA and an increase in MD within exercisers. While this was contrary to our expectations, there have been previous reports of similar findings. Moreover, controls showed greater increases in FA and decreases in MD than exercisers, adding to the existing skepticism of the interpretation that increased FA and decreased MD are always beneficial in aging. However, given the criticisms of the diffusion tensor model regarding crossing fibers, perhaps other metrics that do not have these same weakness are better suited to investigate age-related changes. Finally, we found weak evidence for decreased FD and FDC in exercisers as compared to controls in frontal regions near the cortex. These decreases were negatively correlated with increases in VO_2_peak overall, as well as increases in DSST scores within exercisers. This suggests that the generalized interpretation that higher density and cross-section of white matter fibers are always associated with better outcomes in aging may not be precise enough. Specifically, changes in FD and FDC in deep versus superficial WM may have different functional implications, and these metrics should be investigated in a region-specific manner to understand potentially differentiated biological underpinnings of FD and FDC changes in different brain areas.

## Author contribution statement

SEP assisted with data acquisition, preprocessed imaging data, analyzed the data, interpreted the results, and wrote the manuscript. MMK preprocessed imaging data and revised the manuscript. NCB designed the neuroimaging protocol and revised the manuscript. CM and JP performed physical assessments including cardiopulmonary exercise testing and revised the manuscript. BW designed the physical assessment protocol and revised the manuscript. SK designed the study and revised the manuscript. UL designed the study, interpreted the results, and revised the manuscript. SD designed the study, interpreted the results, and revised the manuscript. EW designed the study, preprocessed imaging data, interpreted the results, and revised the manuscript.

## Declaration of competing interest

The authors declare that they have no known competing financial interests or personal relationships that could have appeared to influence the work reported in this paper.

## Data Availability

Data will be made available on OSF: https://osf.io/y5u24/.

## References

[bib1] Avants B.B., Tustison N.J., Song G., Cook P.A., Klein A., Gee J.C. (2011). A reproducible evaluation of ANTs similarity metric performance in brain image registration. Neuroimage.

[bib2] Avants B.B., Yushkevich P., Pluta J., Minkoff D., Korczykowski M., Detre J., Gee J.C. (2010). The optimal template effect in hippocampus studies of diseased populations. Neuroimage.

[bib3] Barha C.K., Davis J.C., Falck R.S., Nagamatsu L.S., Liu-Ambrose T. (2017). Sex differences in exercise efficacy to improve cognition: a systematic review and meta-analysis of randomized controlled trials in older humans. Front. Neuroendocrinol..

[bib4] Basser P.J., Pierpaoli C. (1996). Microstructural and physiological features of tissues elucidated by quantitative-diffusion-tensor MRI. J. Magn. Reson., Ser. B.

[bib5] Beaulieu C. (2002). The basis of anisotropic water diffusion in the nervous system—a technical review. NMR Biomed..

[bib6] Beck D., de Lange A.-M.G., Maximov I.I., Richard G., Andreassen O.A., Nordvik J.E., Westlye L.T. (2021). White matter microstructure across the adult lifespan: a mixed longitudinal and cross-sectional study using advanced diffusion models and brain-age prediction. Neuroimage.

[bib7] Bendlin B.B., Fitzgerald M.E., Ries M.L., Xu G., Kastman E.K., Thiel B.W., Rowley H.A., Lazar M., Alexander A.L., Johnson S.C. (2010). White matter in aging and cognition: a cross-sectional study of microstructure in adults aged eighteen to eighty-three. Dev. Neuropsychol..

[bib8] Benedict C., Brooks S.J., Kullberg J., Nordenskjöld R., Burgos J., Le Grevès M., Kilander L., Larsson E.-M., Johansson L., Ahlström H., Lind L., Schiöth H.B. (2013). Association between physical activity and brain health in older adults. Neurobiol. Aging.

[bib9] Bennett I.J., Madden D.J. (2014). Disconnected aging: cerebral white matter integrity and age-related differences in cognition. Neuroscience.

[bib10] Burzynska A.Z., Wong C.N., Voss M.W., Cooke G.E., Gothe N.P., Fanning J., McAuley E., Kramer A.F. (2015). Physical activity is linked to greater moment-to-moment variability in spontaneous brain activity in older adults. PLoS One.

[bib11] Choy S.W., Bagarinao E., Watanabe H., Ho E.T.W., Maesawa S., Mori D., Hara K., Kawabata K., Yoneyama N., Ohdake R., Imai K., Masuda M., Yokoi T., Ogura A., Taoka T., Koyama S., Tanabe H.C., Katsuno M., Wakabayashi T. (2020). Changes in white matter fiber density and morphology across the adult lifespan: a cross‐sectional fixel‐based analysis. Hum. Brain Mapp..

[bib12] Clark C.M., Guadagni V., Mazerolle E.L., Hill M., Hogan D.B., Pike G.B., Poulin M.J. (2019). Effect of aerobic exercise on white matter microstructure in the aging brain. Behav. Brain Res..

[bib13] Colcombe S.J., Erickson K.I., Scalf P.E., Kim J.S., Prakash R., McAuley E., Elavsky S., Marquez D.X., Hu L., Kramer A.F. (2006). Aerobic exercise training increases brain volume in aging humans. J. Gerontol. Ser.: Biol. Sci. Med. Sci..

[bib14] Damoiseaux J.S., Smith S.M., Witter M.P., Sanz-Arigita E.J., Barkhof F., Scheltens P., Stam C.J., Zarei M., Rombouts S.A.R.B. (2009). White matter tract integrity in aging and Alzheimer's disease. Hum. Brain Mapp..

[bib15] Dhollander T., Raffelt D., Connelly A. (2016). Proceedings of the 24th Annual Meeting of the International Society of Magnetic Resonance in Medicine.

[bib16] Diedenhofen B., Musch J. (2015). Cocor: a comprehensive solution for the statistical comparison of correlations. PLoS One.

[bib17] Duff E.P., Cunnington R., Egan G.F. (2007). REX: response exploration for neuroimaging Datasets. Neuroinformatics.

[bib18] Düzel S., Drewelies J., Polk S.E., Misgeld C., Porst J., Wolfarth B., Kühn S., Brandmaier A.M., Wenger E. (2022). No evidence for a boost in psychosocial functioning in older age after a 6-months physical exercise intervention. Front. Hum. Neurosci..

[bib19] Erickson K.I., Colcombe S.J., Wadhwa R., Bherer L., Peterson M.S., Scalf P.E., Kim J.S., Alvarado M., Kramer A.F. (2007). Training-induced plasticity in older adults: effects of training on hemispheric asymmetry. Neurobiol. Aging.

[bib20] Erickson K.I., Hillman C., Stillman C.M., Ballard R.M., Bloodgood B., Conroy D.E., Macko R., Marquez D.X., Petruzzello S.J., Powell K.E. (2019). Physical activity, cognition, and brain outcomes: a review of the 2018 physical activity guidelines. Med. Sci. Sports Exerc..

[bib21] Fling B.W., Chapekis M., Reuter-Lorenz P.A., Anguera J., Bo J., Langan J., Welsh R.C., Seidler R.D. (2011). Age differences in callosal contributions to cognitive processes. Neuropsychologia.

[bib22] Genc S., Tax C.M.W., Raven E.P., Chamberland M., Parker G.D., Jones D.K. (2020). Impact of *b*‐value on estimates of apparent fibre density. Hum. Brain Mapp..

[bib23] Geng Z., Liu H., Wang L., Zhu Q., Song Z., Chang R., Lv H. (2016). A voxel-based morphometric study of age- and sex-related changes in white matter volume in the normal aging brain. Neuropsychiatric Dis. Treat..

[bib24] Gow A.J., Bastin M.E., Munoz Maniega S., Valdes Hernandez M.C., Morris Z., Murray C., Royle N.A., Starr J.M., Deary I.J., Wardlaw J.M. (2012). Neuroprotective lifestyles and the aging brain: activity, atrophy, and white matter integrity. Neurology.

[bib25] Guevara M., Guevara P., Román C., Mangin J.-F. (2020). Superficial white matter: a review on the dMRI analysis methods and applications. Neuroimage.

[bib26] Gunning-Dixon F.M., Brickman A.M., Cheng J.C., Alexopoulos G.S. (2009). Aging of cerebral white matter: a review of MRI findings. Int. J. Geriatr. Psychiatr..

[bib27] Harrell F.E.J. (2021). *Hmisc: harrell miscellaneous* (4.6-0). https://CRAN.R-project.org/package=Hmisc.

[bib28] Ho A.J., Raji C.A., Becker J.T., Lopez O.L., Kuller L.H., Hua X., Dinov I.D., Stein J.L., Rosano C., Toga A.W., Thompson P.M. (2011). The effects of physical activity, education, and body mass index on the aging brain. Hum. Brain Mapp..

[bib29] Hong Z., Ng K.K., Sim S.K.Y., Ngeow M.Y., Zheng H., Lo J.C., Chee M.W.L., Zhou J. (2015). Differential age-dependent associations of gray matter volume and white matter integrity with processing speed in healthy older adults. Neuroimage.

[bib30] Hsu J.-L., Leemans A., Bai C.-H., Lee C.-H., Tsai Y.-F., Chiu H.-C., Chen W.-H. (2008). Gender differences and age-related white matter changes of the human brain: a diffusion tensor imaging study. Neuroimage.

[bib31] Jenkinson M., Beckmann C.F., Behrens T.E.J., Woolrich M.W., Smith S.M. (2012). FSL. *NeuroImage*.

[bib32] Jeurissen B., Leemans A., Tournier J.-D., Jones D.K., Sijbers J. (2013). Investigating the prevalence of complex fiber configurations in white matter tissue with diffusion magnetic resonance imaging: prevalence of Multifiber Voxels in WM. Hum. Brain Mapp..

[bib33] Jeurissen B., Tournier J.-D., Dhollander T., Connelly A., Sijbers J. (2014). Multi-tissue constrained spherical deconvolution for improved analysis of multi-shell diffusion MRI data. Neuroimage.

[bib34] Johnson N.F., Kim C., Clasey J.L., Bailey A., Gold B.T. (2012). Cardiorespiratory fitness is positively correlated with cerebral white matter integrity in healthy seniors. Neuroimage.

[bib35] Kassambara A. (2021). https://CRAN.R-project.org/package=rstatix.

[bib36] Kelley S., Plass J., Bender A.R., Polk T.A. (2021). Age-related differences in white matter: understanding tensor-based results using fixel-based analysis. Cerebr. Cortex.

[bib37] Kennedy K.M., Raz N. (2009). Aging white matter and cognition: differential effects of regional variations in diffusion properties on memory, executive functions, and speed. Neuropsychologia.

[bib38] Kerchner G.A., Racine C.A., Hale S., Wilheim R., Laluz V., Miller B.L., Kramer J.H. (2012). Cognitive processing speed in older adults: relationship with white matter integrity. PLoS One.

[bib39] Kirilina E., Helbling S., Morawski M., Pine K., Reimann K., Jankuhn S., Dinse J., Deistung A., Reichenbach J.R., Trampel R., Geyer S., Müller L., Jakubowski N., Arendt T., Bazin P.-L., Weiskopf N. (2020). Superficial white matter imaging: contrast mechanisms and whole-brain in vivo mapping. Sci. Adv..

[bib40] Li Y., Guo T., Guan X., Gao T., Sheng W., Zhou C., Wu J., Xuan M., Gu Q., Zhang M., Yang Y., Huang P. (2020).

[bib41] Liu H., Yang Y., Xia Y., Zhu W., Leak R.K., Wei Z., Wang J., Hu X. (2016). Aging of cerebral white matter. Ageing Res. Rev..

[bib42] Liu Z., Farzinfar M., Katz L.M., Zhu H., Goodlett C., Gerig G., Styner M., Marks B.L. (2012). Automated voxel-wise brain DTI analysis of fitness and aging. Open Med. Imag. J..

[bib43] Lövdén M., Köhncke Y., Laukka E.J., Kalpouzos G., Salami A., Li T.-Q., Fratiglioni L., Bäckman L. (2014). Changes in perceptual speed and white matter microstructure in the corticospinal tract are associated in very old age. Neuroimage.

[bib44] Magistro D., Takeuchi H., Nejad K.K., Taki Y., Sekiguchi A., Nouchi R., Kotozaki Y., Nakagawa S., Miyauchi C.M., Iizuka K., Yokoyama R., Shinada T., Yamamoto Y., Hanawa S., Araki T., Hashizume H., Sassa Y., Kawashima R. (2015). The relationship between processing speed and regional white matter volume in healthy young people. PLoS One.

[bib45] Marks B., Katz L., Styner M., Smith J. (2011). Aerobic fitness and obesity: relationship to cerebral white matter integrity in the brain of active and sedentary older adults. Br. J. Sports Med..

[bib46] Mito R., Raffelt D., Dhollander T., Vaughan D.N., Tournier J.-D., Salvado O., Brodtmann A., Rowe C.C., Villemagne V.L., Connelly A. (2018). Fibre-specific white matter reductions in Alzheimer's disease and mild cognitive impairment. Brain.

[bib47] Nazeri A., Chakravarty M.M., Rajji T.K., Felsky D., Rotenberg D.J., Mason M., Xu L.N., Lobaugh N.J., Mulsant B.H., Voineskos A.N. (2015). Superficial white matter as a novel substrate of age-related cognitive decline. Neurobiol. Aging.

[bib48] Papp K.V., Kaplan R.F., Springate B., Moscufo N., Wakefield D.B., Guttmann C.R.G., Wolfson L. (2014). Processing speed in normal aging: effects of white matter hyperintensities and hippocampal volume loss. Aging Neuropsychol. Cognit..

[bib49] Pierpaoli C., Basser P.J. (1996). Toward a quantitative assessment of diffusion anisotropy. Magn. Reson. Med..

[bib50] Polk S.E., Kleemeyer M.M., Köhncke Y., Brandmaier A.M., Bodammer N.C., Misgeld C., Porst J., Wolfarth B., Kühn S., Lindenberger U., Wenger E., Düzel S. (2022). Change in latent gray-matter structural integrity is associated with change in cardiovascular fitness in older adults who engage in at-home aerobic exercise. Front. Hum. Neurosci..

[bib51] R Core Team (2021). https://www.R-project.org/.

[bib52] Raffelt D.A., Smith R.E., Ridgway G.R., Tournier J.-D., Vaughan D.N., Rose S., Henderson R., Connelly A. (2015). Connectivity-based fixel enhancement: whole-brain statistical analysis of diffusion MRI measures in the presence of crossing fibres. Neuroimage.

[bib53] Raffelt D.A., Tournier J.-D., Rose S., Ridgway G.R., Henderson R., Crozier S., Salvado O., Connelly A. (2012). Apparent Fibre Density: a novel measure for the analysis of diffusion-weighted magnetic resonance images. Neuroimage.

[bib54] Raffelt D.A., Tournier J.-D., Smith R.E., Vaughan D.N., Jackson G., Ridgway G.R., Connelly A. (2017). Investigating white matter fibre density and morphology using fixel-based analysis. Neuroimage.

[bib55] Rau Y.-A., Wang S.-M., Tournier J.-D., Lin S.-H., Lu C.-S., Weng Y.-H., Chen Y.-L., Ng S.-H., Yu S.-W., Wu Y.-M., Tsai C.-C., Wang J.-J. (2019). A longitudinal fixel-based analysis of white matter alterations in patients with Parkinson's disease. Neuroimage: Clinica.

[bib56] Raz N., Lindenberger U., Rodrigue K.M., Kennedy K.M., Head D., Williamson A., Dahle C., Gerstorf D., Acker J.D. (2005). Regional brain changes in aging healthy adults: general trends, individual differences and modifiers. Cerebr. Cortex.

[bib57] Reginold W., Luedke A.C., Itorralba J., Fernandez-Ruiz J., Islam O., Garcia A. (2016). Altered superficial white matter on tractography MRI in Alzheimer's disease. Dementia and Geriatric Cognitive Disorders Extra.

[bib58] Reveley C., Seth A.K., Pierpaoli C., Silva A.C., Yu D., Saunders R.C., Leopold D.A., Ye F.Q. (2015). Superficial white matter fiber systems impede detection of long-range cortical connections in diffusion MR tractography. Proc. Natl. Acad. Sci. USA.

[bib59] Rojas-Vite G., Coronado-Leija R., Narvaez-Delgado O., Ramírez-Manzanares A., Marroquín J.L., Noguez-Imm R., Aranda M.L., Scherrer B., Larriva-Sahd J., Concha L. (2019). Histological validation of per-bundle water diffusion metrics within a region of fiber crossing following axonal degeneration. Neuroimage.

[bib60] RStudio Team (2021). *RStudio: integrated development Environment for R [computer software]*. RStudio. PBC.

[bib61] Sexton C.E., Betts J.F., Demnitz N., Dawes H., Ebmeier K.P., Johansen-Berg H. (2016). A systematic review of MRI studies examining the relationship between physical fitness and activity and the white matter of the ageing brain. Neuroimage.

[bib62] Sexton C.E., Walhovd K.B., Storsve A.B., Tamnes C.K., Westlye L.T., Johansen-Berg H., Fjell A.M. (2014). Accelerated changes in white matter microstructure during aging: a longitudinal diffusion tensor imaging study. J. Neurosci..

[bib63] Smith S.M., Jenkinson M., Johansen-Berg H., Rueckert D., Nichols T.E., Mackay C.E., Watkins K.E., Ciccarelli O., Cader M.Z., Matthews P.M., Behrens T.E.J. (2006). Tract-based spatial statistics: voxelwise analysis of multi-subject diffusion data. Neuroimage.

[bib64] Smith S.M., Jenkinson M., Woolrich M.W., Beckmann C.F., Behrens T.E.J., Johansen-Berg H., Bannister P.R., De Luca M., Drobnjak I., Flitney D.E., Niazy R.K., Saunders J., Vickers J., Zhang Y., De Stefano N., Brady J.M., Matthews P.M. (2004). Advances in functional and structural MR image analysis and implementation as FSL. Neuroimage.

[bib65] Stillman C.M., Esteban-Cornejo I., Brown B., Bender C.M., Erickson K.I. (2020). Effects of exercise on brain and cognition across age groups and health states. Trends Neurosci..

[bib66] Tahedl M. (2018).

[bib67] Tian Q., Erickson K.I., Simonsick E.M., Aizenstein H.J., Glynn N.W., Boudreau R.M., Newman A.B., Kritchevsky S.B., Yaffe K., Harris T.B., Rosano C. (2014). Physical activity predicts microstructural integrity in memory-related networks in very old adults. J. Gerontol. Ser.: Biol. Sci. Med. Sci..

[bib68] Tournier J.-D., Smith R., Raffelt D., Tabbara R., Dhollander T., Pietsch M., Christiaens D., Jeurissen B., Yeh C.-H., Connelly A. (2019). MRtrix3: a fast, flexible and open software framework for medical image processing and visualisation. Neuroimage.

[bib69] Tseng B.Y., Gundapuneedi T., Khan M.A., Diaz-Arrastia R., Levine B.D., Lu H., Huang H., Zhang R. (2013). White matter integrity in physically fit older adults. Neuroimage.

[bib70] Tseng B.Y., Uh J., Rossetti H.C., Cullum C.M., Diaz-Arrastia R.F., Levine B.D., Lu H., Zhang R. (2013). Masters athletes exhibit larger regional brain volume and better cognitive performance than sedentary older adults: MRI Study in Old Athletes' Brains. J. Magn. Reson. Imag..

[bib71] Turken U., Whitfield-Gabrieli S., Bammer R., Baldo J.V., Dronkers N.F., Gabrieli J.D.E. (2008). Cognitive processing speed and the structure of white matter pathways: convergent evidence from normal variation and lesion studies. Neuroimage.

[bib72] Voss M.W., Heo S., Prakash R.S., Erickson K.I., Alves H., Chaddock L., Szabo A.N., Mailey E.L., Wójcicki T.R., White S.M., Gothe N., McAuley E., Sutton B.P., Kramer A.F. (2013). The influence of aerobic fitness on cerebral white matter integrity and cognitive function in older adults: results of a one-year exercise intervention: aerobic Fitness, White Matter, and Aging. Hum. Brain Mapp..

[bib78] Wassenaar T.M., Yaffe K., van der Werf Y.D., Sexton C.E. (2019). Associations between modifiable risk factors and white matter of the aging brain: Insights from diffusion tensor imaging studies. Neurobiology of Aging.

[bib73] Wechsler D. (1981). The psychometric tradition: developing the Wechsler adult intelligence scale. Contemp. Educ. Psychol..

[bib74] Wenger E., Düzel S., Polk S.E., Bodammer N.C., Misgeld C., Porst J., Wolfarth B., Kühn S., Lindenberger U. (2022). *Vamos en bici: Study protocol of an investigation of cognitive and neural changes following language training, physical exercise training, or a combination of both* [Preprint]. BioRxiv.

[bib75] Woolrich M.W., Jbabdi S., Patenaude B., Chappell M., Makni S., Behrens T., Beckmann C., Jenkinson M., Smith S.M. (2009). Bayesian analysis of neuroimaging data in FSL. Neuroimage.

[bib76] Young J., Angevaren M., Rusted J., Tabet N. (2015). Aerobic exercise to improve cognitive function in older people without known cognitive impairment. Cochrane Database Syst. Rev..

[bib77] Zarkali A., McColgan P., Leyland L.-A., Lees A.J., Rees G., Weil R.S. (2020). Fiber-specific white matter reductions in Parkinson hallucinations and visual dysfunction. Neurology.

