# Supplementary material for: Aerobic exercise is associated with region-specific changes in volumetric, tensor-based, and fixel-based measures of white matter integrity in healthy older adults
Source: Neuroimage Rep. 2023 Jan 4;3(1):100155. doi: 10.1016/j.ynirp.2022.100155 (PMC12172729; doi:10.1016/j.ynirp.2022.100155)
Supplement: Multimedia component 1 [file mmc1.docx]

**Supplementary Material**

# Materials and Methods

### Magnetic resonance imaging

#### Preprocessing and calculation of voxel-wise values

T_1_-weighted images were preprocessed using the Computational Anatomy Toolbox 12 (CAT12, Structural Brain Mapping group, Jena University Hospital) in Statistical Parametric Mapping (SPM12, Institute of Neurology). The longitudinal preprocessing pipeline with default parameters was used, in which within-subject inverse-consistent realignment with intra-subject bias correction is first applied to calculate a mean image for each participant before data segmentation. Dartel normalization was then used to estimate spatial normalization parameters based on the segmented images of the within-subject mean image, which were applied to the segmented images at each time point. Nonlinear-only modulation of gray and white matter segmentations was applied. Images were smoothed using an 8-mm full-width half-maximum (FWHM) standard Gaussian kernel.

Diffusion-weighted images were preprocessed using MRtrix (version 3.0_RC3; Tournier et al., 2019), FSL (FMRIB's Software Library, version 6.0.2; Jenkinson et al., 2012; Smith et al., 2004; Woolrich et al., 2009), ANTS (version 2.2.0; Avants et al., 2010, 2011), following the Basic and Advanced Tractography with MRtrix for All Neurophiles (B.A.T.M.A.N.) tutorial (Tahedl, 2018). Images were denoised with a 5 × 5 × 5 patch and Gibb’s ringing artifacts were removed using *dwidenoise* and *mrdegibbs* in MRtrix. Corrections for EPI distortion, B_0_-field inhomogeneity, and eddy-current and movement distortion using *topup* and *eddy_cuda* in FSL with a quadratic spatial model for *eddy*, replacing outliers and saving contrast-to-noise ratio and residual maps. Binary brain masks were generated from each individual’s mean b0 image using *bet* in FSL; these maps were visually inspected and manually adjusted where necessary.

To create FA and MD maps, we followed the TBSS User Guide from FSL (<https://fsl.fmrib.ox.ac.uk/fsl/fslwiki/TBSS/UserGuide>; Smith et al., 2004, 2006). First, FA images were slightly eroded and end slices were zeroed with *tbss_1_preproc*. Nonlinear registration was then run and FA images were aligned to a 1mm^3^ standard space using the JHU ICBM FA 1mm^3^ atlas provided by FSL (<https://identifiers.org/neurovault.image:1402>) with *tbss_2_reg*. Following this, nonlinear transforms generated by the previous step were applied to all subjects to bring them into standard space with *tbss_3_postreg*, resulting in 4D FA data for all subjects. MD maps were generated in a similar manner using *tbss_non_FA*: the nonlinear registration from the FA pipeline was also applied to the MD images and the resulting data were merged into a 4D image. The resulting FA and MD images were then smoothed in SPM using a 4-mm FWHM standard Gaussian kernel for analysis in SPM.

Following preprocessing, FD, log(FC), and FDC were calculated following the “Fibre density and cross-section – Multi-tissue CSD” tutorial from the MRtrix3 documentation (<https://mrtrix.readthedocs.io/en/latest/fixel_based_analysis/mt_fibre_density_cross-section.html>; Tournier et al., 2019). First, average tissue response functions were computed using *dwi2response* and *responsemean.* Multi-shell, multi-tissue CSD was then performed to estimate the FOD using *dwi2fod msmt_csd*. Joint bias field correction and global intensity normalization were run using *mtnormalise.* Intra-subject templates were created from the FOD images from all time points using *population_template*, to which all images within each subject were registered with *mrregister*. An unbiased, study-specific FOD template was then created using 40 randomly selected participants (ten from each intervention group of the parent study) with *population_template*, to which each within-subject FOD template was registered with *mrregister*. A template mask of voxels containing data in all images was created using *mrtransform* and *mrmath*, on which fixel segmentation was performed with fod2fixel and the default threshold of 0.06 to compute a WM template fixel mask. Intra-subject and study-specific warps were then combined using *transformcompose*, and were subsequently applied to the individual unwarped FOD images with *mrtransform* to bring them into common space without FOD reorientation. Each subject’s FOD image was then segmented to estimate fixels and their FD using *fod2fixel*, fixels of the subject FOD images were reoriented in template space using *fixelreorient*, and subject fixels were assigned to template fixels using *fixelcorrespondence* to establish which fixels correspond across subjects, as well as between subject and template fixels. Next, the fiber cross-section metric, FC, was calculated for each subject, as well as the log(FC) metric using *warp2metric* and *mrcalc*, and the combined measure of FDC was calculated by multiplying the FD and FC values from the previous steps using *mrcalc*. Finally, in order to conduct repeated measures ANOVA on the fixel-based metrics in SPM, voxel-wise metrics were calculated for FD, log(FC), and FDC using *fixel2voxel*. For FD and FDC, this was achieved by summing the fixel-wise values across directions to calculate total FD and FDC per voxel; for log(FC), a weighted mean based on the FD values was calculated across directions, resulting in an average voxel-wise log(FC) where the log(FC) value of the direction with the greatest FD was weighted most heavily. These voxel-wise metrics were converted to Nifti format for longitudinal analysis in SPM using *mrconvert*. Finally, the FD, log(FC), and FDC maps were smoothed in SPM using a 10-mm FWHM standard Gaussian kernel.

# References

Avants, B. B., Tustison, N. J., Song, G., Cook, P. A., Klein, A., & Gee, J. C. (2011). A reproducible evaluation of ANTs similarity metric performance in brain image registration. *NeuroImage*, *54*(3), 2033–2044. https://doi.org/10.1016/j.neuroimage.2010.09.025

Avants, B. B., Yushkevich, P., Pluta, J., Minkoff, D., Korczykowski, M., Detre, J., & Gee, J. C. (2010). The optimal template effect in hippocampus studies of diseased populations. *NeuroImage*, *49*(3), 2457–2466. https://doi.org/10.1016/j.neuroimage.2009.09.062

Jenkinson, M., Beckmann, C. F., Behrens, T. E. J., Woolrich, M. W., & Smith, S. M. (2012). FSL. *NeuroImage*, *62*(2), 782–790. https://doi.org/10.1016/j.neuroimage.2011.09.015

Smith, S. M., Jenkinson, M., Johansen-Berg, H., Rueckert, D., Nichols, T. E., Mackay, C. E., Watkins, K. E., Ciccarelli, O., Cader, M. Z., Matthews, P. M., & Behrens, T. E. J. (2006). Tract-based spatial statistics: Voxelwise analysis of multi-subject diffusion data. *NeuroImage*, *31*(4), 1487–1505. https://doi.org/10.1016/j.neuroimage.2006.02.024

Smith, S. M., Jenkinson, M., Woolrich, M. W., Beckmann, C. F., Behrens, T. E. J., Johansen-Berg, H., Bannister, P. R., De Luca, M., Drobnjak, I., Flitney, D. E., Niazy, R. K., Saunders, J., Vickers, J., Zhang, Y., De Stefano, N., Brady, J. M., & Matthews, P. M. (2004). Advances in functional and structural MR image analysis and implementation as FSL. *NeuroImage*, *23*, S208–S219. https://doi.org/10.1016/j.neuroimage.2004.07.051

Tahedl, M. (2018). *B.A.T.M.A.N.: Basic and Advanced Tractography with MRtrix for All Neurophiles*. https://doi.org/10.17605/OSF.IO/FKYHT

Tournier, J.-D., Smith, R., Raffelt, D., Tabbara, R., Dhollander, T., Pietsch, M., Christiaens, D., Jeurissen, B., Yeh, C.-H., & Connelly, A. (2019). MRtrix3: A fast, flexible and open software framework for medical image processing and visualisation. *NeuroImage*, *202*, 116137. https://doi.org/10.1016/j.neuroimage.2019.116137

Woolrich, M. W., Jbabdi, S., Patenaude, B., Chappell, M., Makni, S., Behrens, T., Beckmann, C., Jenkinson, M., & Smith, S. M. (2009). Bayesian analysis of neuroimaging data in FSL. *NeuroImage*, *45*(1), S173–S186. https://doi.org/10.1016/j.neuroimage.2008.10.055
